# Heavy metals uptake by the global economic crop (*Pisum sativum* L.) grown in contaminated soils and its associated health risks

**DOI:** 10.1371/journal.pone.0252229

**Published:** 2021-06-04

**Authors:** Tarek M. Galal, Loutfy M. Hassan, Dalia A. Ahmed, Saad A. M. Alamri, Sulaiman A. Alrumman, Ebrahem M. Eid

**Affiliations:** 1 Faculty of Science, Botany and Microbiology Department, Helwan University, Cairo, Egypt; 2 Department of Biology, College of Sciences, Taif University, Taif, Saudi Arabia; 3 Faculty of Science, Botany Department, Tanta University, Tanta, Egypt; 4 Biology Department, College of Science, King Khalid University, Abha, Saudi Arabia; 5 Faculty of Science, Botany Department, Kafrelsheikh University, Kafr El-Sheikh, Egypt; Universidade de Coimbra, PORTUGAL

## Abstract

The aim of the present investigation was to determine the concentration of heavy metals in the different organs of *Pisum sativum* L. (garden pea) grown in contaminated soils in comparison to nonpolluted soils in the South Cairo and Giza provinces, Egypt, and their effect on consumers’ health. To collect soil and plant samples from two nonpolluted and two polluted farms, five quadrats, each of 1 m^2^, were collected per each farm and used for growth measurement and chemical analysis. The daily intake of metals (DIM) and its associated health risks (health risk index (HRI) were also assessed. The investigated heavy metals were cadmium (Cd), arsenic (As), chromium (Cr), copper (Cu), nickel (Ni), iron (Fe), manganese (Mn), zinc (Zn), silver (Ag), cobalt (Co) and vanadium (V). Significant differences in soil heavy metals, except As, between nonpolluted and polluted sites were recorded. Fresh and dry phytomass, photosynthetic pigments, fruit production, and organic and inorganic nutrients were reduced in the polluted sites, where there was a high concentration of heavy metals in the fruit. The bioaccumulation factor for all studied heavy metals exceeded 1 in the polluted sites and only Pb, Cu and Mn exceeded 1 in the nonpolluted sites. Except for Fe, the DIM of the studied heavy metals in both sites did not exceed 1 in either children or adults. However, the HRI of Pb, Cd, Fe, and Mn in the polluted plants and Pb in the nonpolluted ones exceeded 1, indicating significant potential health risks to consumers. The authors recommend not to eat garden peas grown in the polluted sites, and farmers should carefully grow heavy metals non-accumulating food crops or non-edible plants for other purposes such as animal forages.

## Introduction

Vegetables are edible plants that store reserve food materials in their roots, stem, leaves, and/or fruits, where essential dietary elements, including iron, calcium, vitamins, protein, and other nutrients, are contained [1]. These elements are the main construction components of the human body that help in the formation of bones, teeth, hair, and nails and protect the human body from various diseases [2]. They also act as protecting agents for acidic materials produced during the digestion process. Vegetables are the main source of minerals, vitamins, and fibers and have useful antioxidative effects [3]. A diet rich in vegetables has been reported to diminish the risk of heart diseases, as well as gastric, lung, and esophageal cancers; however, they may pose a hazard to human health when contaminated with heavy metals [4].

Chaotic and rapid industrial and urban development is one of the main reasons for elevated levels of environmentally toxic heavy metals in developing countries such as Egypt [5]. In addition, Egyptian environments polluted by heavy metals commonly result in environmental toxicity [6]. Besides, heavy metal contamination of agricultural soils and crops is particularly worse in developing industrialized countries such as Egypt, due to extensive use of untreated industrial wastewater [7]. In contaminated agricultural soils, heavy metals may affect the quality of vegetable crops, and accumulation of these heavy metals by crop plants can have deleterious effects on human health [8]. Furthermore, the uptake and accumulation of high levels of heavy metals by crop plants grown in polluted soils represents the main pathway for bioaccumulation of these heavy metals into the food web [8]. Other sources of heavy metals in irrigated agriculture include manures, fertilizers, and pesticides, as well as airborne contamination from car traffic [9].

Heavy metals are of major concern due to their toxic impacts on the environment [10]. Worldwide, they are the most serious issue in vegetable growth and yield in polluted agricultural lands [11]. Heavy metals can cause soil degradation and hence reduce vegetable quality, productivity, and safety leading to unsustainability of vegetable production [12]. Vegetables grown on or near polluted lands have a high ability to accumulate heavy metals from the environment [7]. The accumulation of heavy metals by a plant depends mainly on the plant species, phenology, heavy metal type, climate, and soil quality [13].

The accumulation of heavy metals in contaminated vegetables may cause a direct threat to human health [14]. Food pollution by these heavy metals represents one of the main aspects of food quality assurance [15]. The accumulation of these metals in vegetable crops represents an indirect pathway for their incorporation into the human food chain, and the transfer of these heavy metals through crop plants cultivated in polluted soil may pose a possible risk to human health [16]. The health risks depend on the degree of environment pollution with heavy metals, the types of vegetables cultivated, and the consumption rate [9]. According to Dong et al. [17], cultivating vegetables in contaminated agricultural soils causes chronic toxicity risks of toxic metals, which potentially affect human health. Metal toxicity in leguminous plants can cause chlorosis, diminished plant growth, yield loss, metabolic disorders, deficiency of nutrient uptake, and reduced atmospheric nitrogen fixation ability [14,18].

Soil has an important role in food safety since it determines the type of producer, which is the base of the food chain; however, there is a lack of adequate data and reliability in studies on the quality of soil resources and their associated health risks [19]. Excessive accumulation of heavy metals by crops from agricultural soils results in soil pollution and poor food quality, and thus it is necessary to improve food quality by taking into consideration the incorporation of heavy metals in the food web through plant absorption [20]. An increased emphasis on food safety has attracted the attention of many researchers to the risks associated with the consumption of polluted vegetables [21].

*Pisum sativum* L. (the garden pea) is an important commercial legume crop grown in temperate and semitropical regions [22]. In Egypt, the total area devoted to dry pea seed production is 9496 acres and produces a total yield of 7000 tons [23]. Its mature green seeds are a rich source of vitamins and proteins (20–22%/seed) in developing countries [18,24]. *P*. *sativum* is cultivated as a main food crop for Egyptians and for exportation [25], consequently this crop must be produced with high quality. Thus, the results of this study are worldwide relevant. According to the authors’ knowledge, so far, no studies have been carried out in Egypt on *P*. *sativum* grown at polluted sites in comparison with the reference (nonpolluted) sites. Therefore, the aim of the present investigation was to determine the accumulation potential of heavy metals in the different organs (root, leaf, and fruit) of *P*. *sativum* cultivated in polluted soils in comparison to nonpolluted soils in the South Cairo Province, Egypt, and to assess the associated impact of these accumulated heavy metals on the health of the public consumers.

## Materials and methods

### Sampling sites

Plant sampling was carried out through two farms in nonpolluted (29°44′38.82″ N and 31°153.00″ E) and two others in polluted (29°44′45.47″ N and 31°17′46.56″ E) sites, located in the South Cairo and Giza Provinces, respectively, during the winter season of 2017. The owner of the farms gave us the permission to conduct the study on these sites. Each farm has an area of approximately three acres. Nonpolluted farms received clean water from the Nile River tributaries, while polluted farms received industrial waste (National Cement Company, Egyptian Iron and Steel, Bricks factories, and Helwan Fertilizer Company) and municipal discharge. These wastes mainly constitute hydrocarbons, nutrients, and heavy metals. The agricultural lands of the study sites, which extends along the River Nile, were characterized by Alluvial soils. The prevailing climate of the study area showed that the mean annual rainfall was 1.67–2.13 mm/year, while the mean annual temperature was 21.08°C, and the annual mean relative humidity was 52.68–56.08%.

### Plant sampling and analysis

At each farm, 5 quadrats (each of 1 m^2^) were randomly selected to represent the growth of *P*. *sativum*. From each quadrat, individual pea plants were harvested, separated into roots, shoots (stems + leaves), and fruits, weighed to determine the fresh weight, packed in polyethylene bags, and transferred to the laboratory. Plant samples were washed twice with tap water and then with distilled water, followed by oven-drying at 105°C until they reached a constant weight, to determine the dry weight (kg/acre) and the plant production [26], and then ground into powder using a metal-free plastic mill for nutrient analysis. For pigment analysis, three pea leaves from each quadrat in the nonpolluted and polluted sites were collected and mixed to make three composite samples. Chlorophyll and carotenoids were extracted from the definite fresh weight of leaves (approximately 2 g) in 50% (v/v) acetone in complete darkness and kept overnight at 4°C, then taken and measured using a spectrophotometer against a blank of aqueous acetone at three wavelengths (453, 644, and 663 nm). The concentration of each pigment fraction in mg/l was calculated using the following equations [27]:

Chlorophylla=(10.3×E663)–(0.918×E644)

Chlorophyllb=(19.7×E644)–(3.87×E663)

Carotenoids=(4.2×E453)–((0.0264×Chlorophylla)+(0.426×Chlorophyllb))

The values were then conveyed as mg/g fresh weight. Total soluble carbohydrates were estimated by the anthrone–sulfuric acid method [28], while the total soluble proteins were measured spectrophotometrically by the Bio-Rad protein assay [29]. Heavy metals and nutrient (N, P, and K) concentrations of the different plant organs were extracted using the mixed acid digestion method. A ground sample of 1 g was digested in 20 mL of a tri-acid mixture of HNO_3_:H_2_SO_4_:HClO_4_ (5:1:1, *v/v/v*) until a transparent color appeared, then the digested plant was filtered and diluted with double-distilled water to 25 mL [30]. Twelve heavy metals (Ag, As, Cd, Co, Cr, Cu, Fe, Mn, Ni, Pb, V, and Zn) and K were determined by atomic absorption spectrometry (Shimadzu AA-6300; Shimadzu Co. Ltd., Kyoto, Japan), while total P was determined using a spectrophotometer via the ammonium molybdate method. Total N was determined using a CHN Elemental Analyzer (Yanako CHN Corder MT-5, Yanaco Apparatus Development Laboratory Co. Ltd., Kyoto, Japan). All procedures are outlined in the study of Allen [26]. Atomic absorption spectrometry was calibrated by standard solutions, which contain known concentrations of each element. Standard solutions were prepared by diluting available high-purity stock solutions (BDH) [26].

### Soil sampling and analysis

Three composite soil samples were collected from the profiles of 0–20 cm from each farm. Soil water extracts (1:5, *w/v*) were prepared to determine the soil salinity (μS/cm) using an electrical conductivity meter (Corning Model 311, Corning Incorporated, New York, USA), and soil reaction using a pH meter (ICM Model B-213, ICM, Hillsboro, USA). N, P, K, and the 12 heavy metals were extracted and determined with the methods used for plant samples.

### Quality assurance and quality control

A certified reference material (SRM 1573a, tomato leaves) was used to verify the accuracy of the heavy metal determinations. This reference material was digested and analyzed using the same methods applied to the *P*. *sativum* samples. Heavy metal digestions and measurements were performed in triplicate. Accuracy was determined by comparing the measured concentration with the certified value, and the result was expressed as a percentage. The recovery rates ranged from 95 to 104% for SRM 1573a. The detection limits of heavy metals (in μg/l) were as follows: 3.0 for Ag, 5.0 for Fe; 1.5 for As, Cu, Mn and Zn; 15.0 for Pb and Cd; 9.0 for Co; 3.0 for Cr, 2.0 for V and 6.0 for Ni. The detection limits for all heavy metals were established on a 95% confidence level (3 standard deviations).

### Data analysis

The soil pollution by each metal was assessed using the pollution load index (PLI), calculated as follows [31]:

PLI=concentrationofheavymetalinpollutedsoils/concentrationofheavymetalinnonpollutedsoils

The significance of the variation in soil and plant variables between the nonpolluted and polluted sites was tested using a paired-samples *t*-test. Before performing two-way analysis of variance (ANOVA-2), the data were tested for their normality of distribution and homogeneity of variance, and when necessary, the data were log-transformed. ANOVA-2 was used to assess the significant variation in the nutrients and heavy metals in the different plant organs between the nonpolluted and polluted sites. Duncan’s multiple range test at *p* < 0.05 was used to identify significant differences between means. Statistical analyses were carried out using SPSS software [32]. The bioaccumulation factor (BF), measuring the plant’s ability to accumulate a specific metal in relation to its concentration in the soil, was calculated as follows: *BF* = Croot/Csoil, where C_root_ and C_soil_ are the concentrations of heavy metals in the roots and soil. The translocation factor (TF), which assesses the relative translocation of heavy metals from the roots to shoots of a plant, was calculated as *TF* = Cshoot/Croot, where C_shoot_ and C_root_ are the concentrations of heavy metals in the plant’s shoots and roots [9].

A health risk assessment of any pollutant requires an estimation of the level of exposure by noticing methods of exposure to the target organisms. The daily intake of metals (DIM) was measured as the average consumption of polluted plants for both adults and children [33]. *DIM* = (Cmetal × Cfactor × Dfood intake)/Baverage weight, where C_metal_ is the metal concentration in the plant (mg/kg), C_factor_ is a conversion factor, D_food intake_ is the daily intake of vegetable, and B_average weight_ is the Egyptian average body weight. The conversion factor (0.085) was used to convert fresh weight to dry weight [34]. The average daily intake of metal for children and adults is 0.345 and 0.232 kg/person/day, while their average body weights are 32.7 and 55.9 kg, respectively [35]. Moreover, the health risk index (HRI) for the local inhabitants consuming contaminated plants was calculated as the ratio of the assessed crop exposure and the reference oral dose [36]. An HRI value greater than 1 is a danger for human health [37] and may cause a health risk for the consumers.

## Results

### Soil analysis

Significant differences for all soil variables (except As) were recognized between the nonpolluted and polluted sites (**Table 1**). The polluted soils had a higher pH (7.6), salinity (5.8 μS/cm), and heavy metal concentrations, but lower contents of N (49.7 mg/kg), P (6.9 mg/kg), and K (35.4 mg/kg) than the nonpolluted soils. The pollution load indexes (PLI) of the investigated heavy metals (except Cr = 0.8) were greater than 1. Based on the PLI, heavy metals were arranged as Pb (92.1) > As (61.0) > V (21.8) > Zn (20.9) > Fe (13.4) > Cu (7.6) > Ag (7.5) > Co (3.8) > Cd (2.2) > Mn (1.9) > Ni (1.3) > Cr (0.8). Moreover, the concentration of all the investigated heavy metals (except Cr, Cu, and Ni) were above the tolerable limits.

**Table 1:** Mean ± standard error of soil characteristics and pollution load index (PLI) of *Pisum sativum* grown in the nonpolluted and polluted sites.

Soil characters	Sites		Tolerable Limits WHO [38]	PLI
	Non-polluted	polluted		
pH	6.4 ± 0.01	7.6 ± 0.04	-	-
EC (μS/cm)	2.6 ± 0.03	5.8 ± 0.05	-	-
Nutrient (mg/kg)				
N	292.8 ± 1.70	49.7 ± 1.50	-	-
P	17.9 ± 0.03	6.9 ± 0.20	-	-
K	450.3 ± 1.50	35.4 ± 0.90	-	-
Heavy metal (mg/kg)				
Pb	0.6 ± 0.01	50.7 ± 1.50	0.01–50	92.1 ± 3.18
Cd	0.2 ± 0.01	20.4 ± 0.02	0.02–0.7	2.2 ± 0.10
As	0.01 ± 0.00	0.6 ± 0.20	0.001	61.0 ± 5.14
Cr	0.5 ± 0.01	0.4 ± 0.02	5–30	0.8 ± 0.03
Cu	2.1 ± 0.06	16.3 ± 0.60	0.27–100	7.6 ± 0.12
Ni	0.5 ± 0.01	0.6 ± 0.01	5.0	1.3 ± 0.05
Fe	12.8 ± 0.02	171.0 ± 2.70	0.15–7	13.4 ± 0.22
Mn	31.4 ± 0.20	60.0 ± 0.90	20.0	1.9 ± 0.03
Zn	3.5 ± 0.01	73.0 ± 4.40	10–50	20.9 ± 4.24
Ag	0.1 ± 0.00	0.6 ± 0.02	0.01	7.5 ± 1.13
Co	0.2 ± 0.00	0.9 ± 0.04	0.02	3.8 ± 0.22
V	0.04 ± 0.00	0.9 ± 0.03	0.001	21.8 ± 2.25

** *p* < 0.01

*** *p* < 0.001; *ns*, not significant (i.e., *p* > 0.05).

### Phytomass

**Fig 1:**
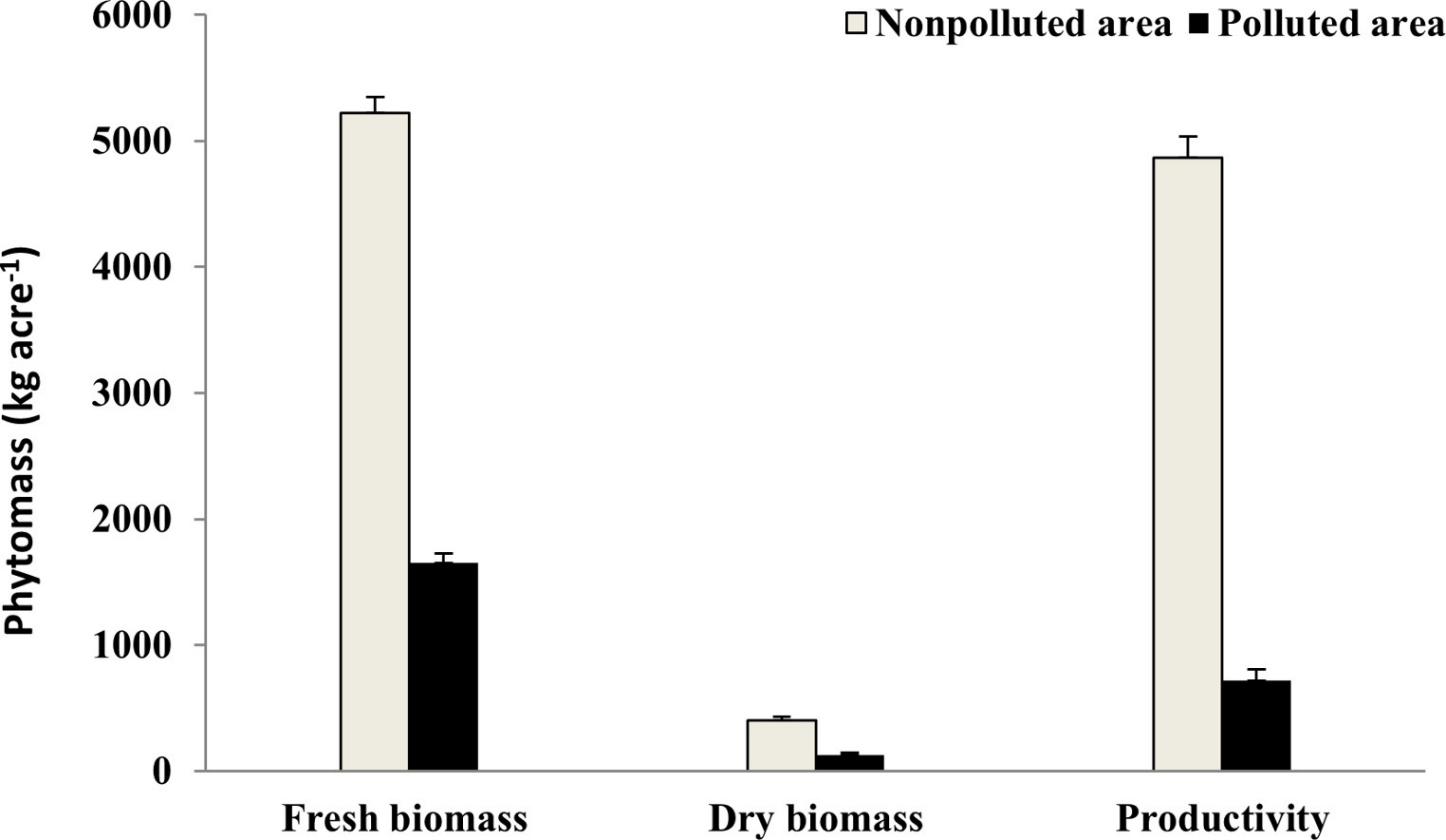
Phytomass and production (kg/acre) of *Pisum sativum* cultivated in nonpolluted and polluted soils. ** *p* < 0.01, *** *p* < 0.001. The standard errors of the means were indicated by vertical bars.

A significant difference was detected for the fresh and dry phytomass, as well as fruit production, between nonpolluted and polluted sites (**Fig 1**). In the polluted soils, the fresh phytomass of *P*. *sativum* was greatly reduced from 5223 to 1654 kg/acre, while the dry phytomass was decreased from 404 to 126 kg/acre. In addition, the fruit production was reduced by 85.2% under pollution stress.

### Plant analysis

**Fig 2:**
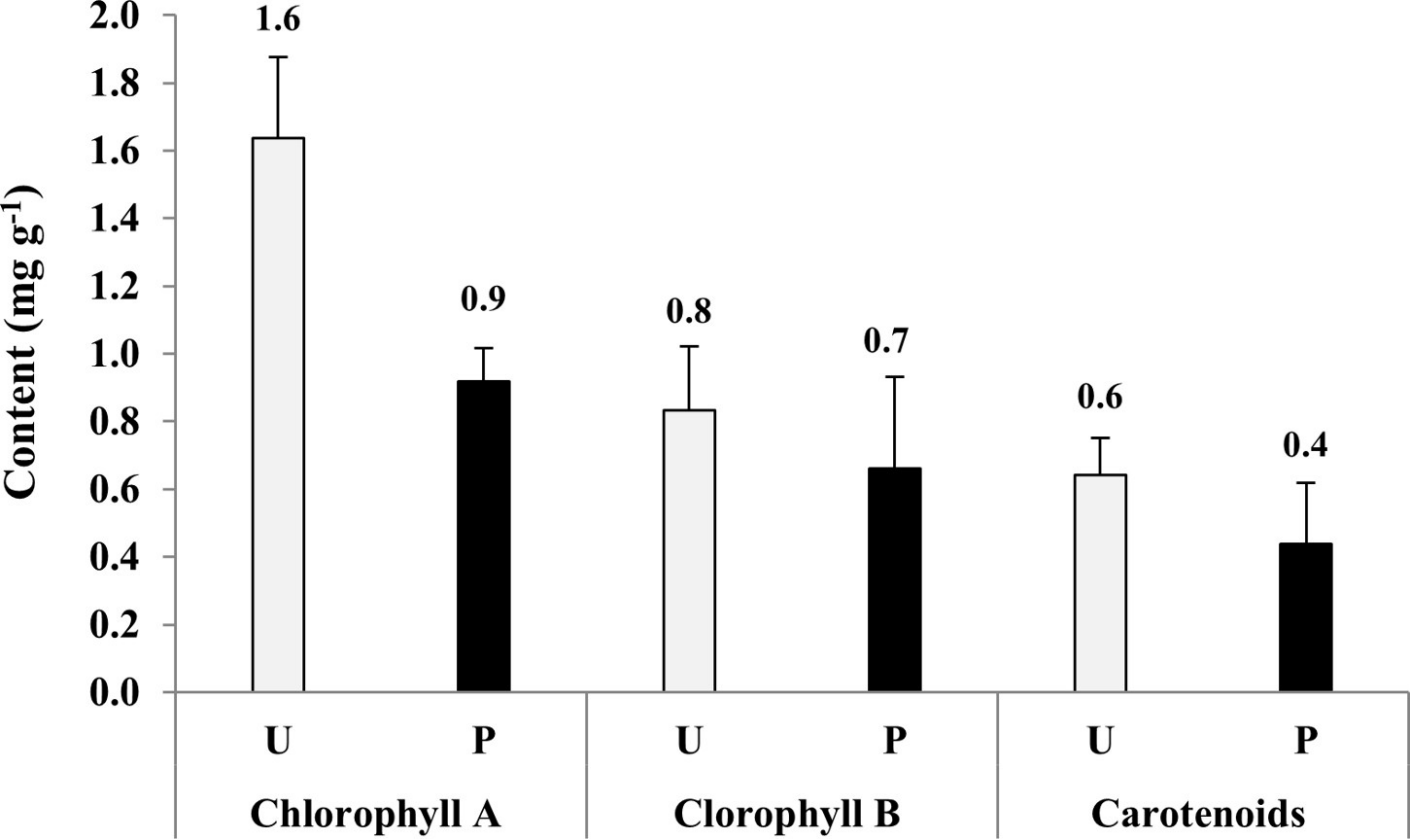
Pigment analysis of the leaves of *Pisum sativum* grown in nonpolluted (Non) and polluted (P) sites. * *p* < 0.05. The standard errors of the means were indicated by vertical bars.

The analysis of *P*. *sativum* leaves indicated a significant reduction in chlorophyll a and a nonsignificant reduction in chlorophyll b and carotenoids in the polluted sites (**Fig 2**). Chlorophyll a was reduced by 43.8%, while chlorophyll b and carotenoids were reduced by 12.5% and 33.3%, respectively. The nutrient contents of the above- and below-ground parts of *P*. *sativum* showed a significant reduction under pollution stress (**Table 2**). The highest carbohydrate and protein contents (14.8% and 14.7%, respectively) were recorded in the nonpolluted plant leaves, while the lowest (10.7% and 10.2%) were recorded in the polluted roots. The highest value of total N (2.3%) was recorded in the nonpolluted leaves, while P and K had the highest values (1.7% and 22.3 mg/kg) in the nonpolluted roots. Meanwhile, N, P, and K had the lowest values (1.6%, 0.7%, and 13.4 mg/kg, respectively) in the polluted roots. Moreover, the highest accumulation of heavy metals was in the below-ground parts of *P*. *sativum* cultivated in the polluted sites. The concentrations of Fe were 2201.3 and 2744.8 mg/kg and of Cd were 1104.0 and 1178.5 mg/kg in the plant shoots and roots, respectively.

**Table 2:** Organic and inorganic nutrient and heavy metal concentrations (mean ± standard deviation) in the shoots and roots of *Pisum sativum* grown in unpolluted and polluted soils.

Parameter	Unpolluted sites		Polluted sites	
	Shoot	Root	Shoot	Root
	Organic nutrients			
Carbohydrates (%)	14.8 ± 1.39^c^	13.0 ± 0.18b^c^	11.4 ± 0.72^ab^	10.7 ± 0.88^a^
Proteins (%)	14.7 ± 1.40^b^	10.4 ± 1.50^a^	14.5 ± 1.74^b^	10.2 ± 1.80^a^
	Inorganic nutrients			
N (%)	2.3 ± 0.22^b^	1.7 ± 0.24^a^	2.3 ± 0.28^b^	1.6 ± 0.29^a^
P (%)	1.5 ± 0.30^bc^	1.7 ± 0.16^c^	1.2 ± 0.16^b^	0.7 ± 0.10^a^
K (mg/kg)	18.5 ± 0.62^b^	22.3 ± 1.99^c^	14.3 ± 0.17^a^	13.4 ± 0.18^a^
	Heavy metals			
Pb (mg/kg)	8.9 ± 1.76^a^	1.2 ± 0.43^a^	142.8 ± 15.67^b^	181.7 ± 10.75^c^
Cd (mg/kg)	0.6 ± 0.14^a^	1.3 ± 0.25^a^	1104.0 ± 3.97^b^	1178.5 ± 6.87^c^
As (mg/kg)	0.04 ± 0.01^a^	0.1 ± 0.03^ab^	0.2 ± 0.05^b^	0.4 ± 0.03^c^
Cr (mg/kg)	0.7 ± 0.06a	1.4 ± 0.29^a^	26.7 ± 8.61^b^	40.3 ± 11.09^c^
Cu (mg/kg)	0.4 ± 0.14^a^	0.7 ± 0.29^a^	14.3 ± 4.75^b^	33.2 ± 10.02^c^
Ni (mg/kg)	1.0 ± 0.04^a^	6.7 ± 0.98^a^	28.8 ± 5.86^b^	46.3 ± 3.88^c^
Fe (mg/kg)	583.5 ± 45.27^a^	658.5 ± 31.25^a^	2201.3 ± 125.62^b^	2744.8 ± 49.9^c^
Mn (mg/kg)	10.9 ± 1.94^a^	9.0 ± 6.31^a^	65.0 ± 3.12^b^	206.5 ± 21^c^
Zn (mg/kg)	3.0 ± 4.35^a^	8.1 ± 0.88^a^	49.8 ± 5.30^b^	91.8 ± 4.37^c^
Ag (mg/kg)	0.6 ± 0.07^a^	0.9 ± 0.03^b^	3.0 ± 0.13^c^	4.4 ± 0.13^d^
Co (mg/kg)	0.5 ± 0.05^a^	1.1 ± 0.07^b^	2.7 ± 0.25^c^	4.5 ± 0.18^d^
V (mg/kg)	0.03 ± 0.01^a^	0.1 ± 0.01^b^	0.2 ± 0.0^c^	0.3 ± 0.03^d^

Means in the same row with different letters are significant according to Duncan’s multiple range test.

### Heavy metals in the fruits

There was a high accumulation of heavy metals in the fruits of *P*. *sativum* plants cultivated in the polluted farms (**Table 3**). The concentration of most of the heavy metals in the fruit tissues exceeded 80% in the polluted farms. The Fe concentration exceeded 1000 mg/kg in the plants cultivated in the polluted sites. The order of heavy metal concentrations in the fruits from the plants grown in the polluted sites was Fe > Cd > Pb > Mn > Zn > Ni > Cr > Cu > Co > Ag > As = V; meanwhile, in the nonpolluted plants, it was Fe > Mn > Pb > Zn > Ag and Co > Ni > Cr > Cd > Cu > As = V.

**Table 3:** Heavy metal concentrations (mean ± standard deviation) in the fruits of *Pisum sativum* grown in nonpolluted and polluted soils. Difference = ((Cp–Cn)/Cp) × 100, where Cp and Cn are the metal concentrations in the polluted and nonpolluted soils, respectively.

Heavy metals (mg/kg)	Sites		Difference (%)	*t-*value
	Non-polluted	Polluted		
Pb	8.9 ± 1.76	121.0 ± 1.32	92.6 ± 22.1	78.7***
Cd	2.6 ± 0.14	1025.0 ± 1.00	99.7 ± 19.6	227.9***
As	0.03 ± 0.00	0.1 ± 0.01	50.0 ± 8.7	12.1**
Cr	0.9 ± 0.33	19.8 ± 0.76	95.6 ± 14.3	46.2***
Cu	0.4 ± 0.14	12.6 ± 0.40	96.7 ± 23.6	51.4***
Ni	0.9 ± 0.38	24.7 ± 0.76	96.3 ± 20.2	37.7***
Fe	583.5 ± 45.27	2098.0 ± 24.02	72.2 ± 16.6	47.4***
Mn	10.9 ± 1.94	61.6 ± 0.79	82.3 ± 18.6	43.0***
Zn	3.0 ± 4.35	25.8 ± 1.53	88.5 ± 19.5	7.0*
Ag	1.5 ± 0.00	2.2 ± 0.13	30.2 ± 6.3	8.5*
Co	1.5 ± 0.00	2.3 ± 0.85	35.6 ± 5.4	1.7^ns^
V	0.03 ± 0.00	0.1 ± 0.00	50.0 ± 4.3	19.0**

* *p* < 0.05

** *p* < 0.01

*** *p* < 0.001

*ns*, not significant (i.e., *p* > 0.05).

### Heavy metal bioaccumulation and translocation

The bioaccumulation factor (BF) of *P*. *sativum* for the studied heavy metals was greater than 1 in the polluted sites, while the translocation factor (TF) did not exceed 1 in either the nonpolluted or polluted sites (**Table 4**). It was found that Cd had the highest BF (242.9) in the polluted sites, followed by Cr (96.9) and Ni (72.4), while Mn had the highest BF (3.5) in the nonpolluted sites, followed by Cu (3.2) and Pb (2.2). Besides, the TF of heavy metals arranged as Fe (0.80) > Pb (0.79) > Ag (0.67) > Cr (0.66) > Ni (0.62) > Co (0.61) > V (0.60) > Cd (0.58) > Zn (0.54) > Cu (0.43) > As (0.41) > Mn (0.31).

**Table 4:** Bioaccumulation (BF) and translocation (TF) factors of heavy metals in *Pisum sativum* grown in nonpolluted and polluted soils.

Heavy metal	Non-polluted sites		Polluted sites	
	BF	TF	BF	TF
Pb	2.22 ± 0.32	0.73 ± 0.12	3.59 ± 0.66	0.79 ± 0.08
Cd	0.14 ± 0.04	0.49 ± 0.06	442.93 ± 32.82	0.58 ± 0.03
As	0.10 ± 0.01	0.42 ± 0.08	4.60 ± 1.03	0.41 ± 0.11
Cr	0.36 ± 0.06	0.49 ± 0.09	96.88 ± 12.63	0.66 ± 0.04
Cu	3.21 ± 0.11	0.63 ± 0.11	2.03 ± 0.45	0.43 ± 0.02
Ni	0.08 ± 0.01	0.16 ± 0.08	72.40 ± 9.42	0.62 ± 0.11
Fe	0.02 ± 0.01	0.89 ± 0.15	16.05 ± 3.21	0.80 ± 0.13
Mn	3.49 ± 0.18	0.12 ± 0.03	3.44 ± 0.86	0.31 ± 0.06
Zn	0.43 ± 0.08	0.37 ± 0.08	1.26 ± 0.09	0.54 ± 0.10
Ag	0.09 ± 0.02	0.62 ± 0.12	7.33 ± 1.01	0.67 ± 0.014
Co	0.23 ± 0.03	0.43 ± 0.03	4.99 ± 0.96	0.61 ± 0.11
V	0.61 ± 0.09	0.50 ± 0.4	10.38 ± 1.78	0.60 ± 0.09

### Daily intake of metals (DIM)

The DIM of heavy metals (except Fe and Cd) in the nonpolluted and polluted sites did not exceed 1 in either children or adults (**Table 5**). The contribution of *P*. *sativum* cultivated in the polluted soil to the dietary intake of Cd (mg/individual.day) was 1.07 for both children and adults, and the contribution of Fe was 1.3 mg/individual.day for children and 1.1 mg/individual.day for adults. The DIM sequence in the nonpolluted sites was Fe > Mn > Pb > Zn > Ag = Co > Cr = Ni > Cd > Cu >As = V, and in the polluted sites, it was Fe > Cd > Pb > Mn > Ni > Zn = Cr > Ag = Co > As = V.

**Table 5:** Daily intake of metals (mg/kg body weight/day) by adults and children for individual heavy metals in *Pisum sativum* grown in nonpolluted and polluted soils.

Heavy metal	Non-polluted sites		Polluted sites	
	Adult	Child	Adult	Child
Pb	0.005	0.005	0.06	0.07
Cd	0.0003	0.0004	1.07	1.07
As	0.00002	0.00002	0.00003	0.00004
Cr	0.0005	0.0005	0.01	0.01
Cu	0.0002	0.0003	0.007	0.008
Ni	0.0005	0.0006	0.01	0.01
Fe	0.3	0.4	1.1	1.3
Mn	0.006	0.007	0.03	0.04
Zn	0.002	0.002	0.01	0.04
Ag	0.0008	0.0009	0.001	0.001
Co	0.0008	0.0009	0.001	0.001
V	0.00002	0.00002	0.00003	0.00004

### Assessment of health risks

The health risk index (HRI) indicated that Pb in the nonpolluted sites and Pb, Cd, Fe, and Mn in the polluted sites have the greatest potential to cause health risks to public consumers, and the HRI of these metals for children and adults exceeded unity (**Table 6**). In the polluted sites, the HRI of Cd (60 and 70) was the highest, followed by that of Pb (60 and 70), Mn (2.1 and 2.9), and Fe (1.8 and 1.9) for both adults and children. In contrast, the HRI of Pb in the nonpolluted sites was 5 for both adults and children.

**Table 6:** Health risk index (HRI) and oral reference dose (RfD) of heavy metals for adults and children via the intake of heavy metals in *Pisum sativum* grown in nonpolluted and polluted sites.

Heavy metal	Non-polluted site		Polluted site		RfD (mg/kg/day)
	Adult	Children	Adult	Children	
US-EPA [39]					
Pb	5	5	60	70	0.001
Cd	0.3	0.4	70	70	0.001
As	0.07	0.07	0.1	0.13	0.0003
Cr	0.0003	0.0003	0.007	0.007	1.500
Co	0.019	0.021	0.023	0.023	0.043
WHO/FAO [40]					
Cu	0.005	0.008	0.175	0.2	0.040
Fe	0.43	0.57	1.8	1.9	0.700
Mn	0.43	0.5	2.14	2.9	0.014
Zn	0.007	0.007	0.033	0.13	0.300
US-EPA [41]					
Ni	0.03	0.03	0.5	0.5	0.020
WHO/FAO [42]					
V	0.00001	0.00001	0.00002	0.00002	1.800

## Discussion

Plants cultivated in heavy-metal-polluted soils, resulting from the increase in anthropogenic and geologic activities, show reduced growth due to changes in their biochemical and physiological activities [43]. In the present study, a remarkable decrease in N, P, and K in the soil and plants (roots and shoots) of the polluted sites was recorded. In most agricultural conditions, the availability of usable nitrogen is a common limiting factor of high growth, as nitrogen is important for vegetative growth and assimilation of amino acids, and it is a constituent of chlorophyll. In addition, a suitable amount of P plays an important role in plants yielding more fruit and having healthier shoots and root systems, and plants with sufficient P may mature much faster than those without phosphorus. An inadequate supply can cause green and purple discoloration, wilting, and small flowers and fruit. K helps plants use water efficiently, preventing many diseases and heat damage, and aids the cycle of nutrients through the leaves, roots, and stems [44].

In this study, the concentration of heavy metals was higher in polluted soils than in nonpolluted soils, which coincides with the results of Galal [6], and Shehata and Galal [9]. Based on the Environmental Quality Standards set by the US Environmental Protection Agency [37], the polluted and nonpolluted soils in the present study were within the safe level. This could be due to the incessant removal of heavy metals by the crops cultivated in these sites and the leaching of heavy metals into the soil’s deeper layer [14]. The values of all of the metals determined were above the tolerable limits recommended by the World Health Organization [38], except for Cr (0.4 mg/kg), Cu (16.3 mg/kg), and Ni (0.6 mg/kg), which were below the standard maxima of 30, 0.27−100.0, and 5.0 mg/kg, respectively.

According to Borah and Devi [22], heavy metals affect the growth performance of *P*. *sativum* by decreasing its biomass, yield, and photosynthetic pigment (chlorophyll a and b) in plants grown in wastewater-irrigated soil. In our study, a reduction in the fresh and dry phytomass and productivity of *P*. *sativum* cultivated in the polluted soils was recorded. In addition, the contents of chlorophyll a and b were also reduced, which may have led to a decrease in *P*. *sativum* productivity. According to Mansur and Garba [45], heavy metal concentrations affect soil fertility, as they reduce the nutrient (e.g., K and P) availability by forming complexes (e.g., lead phosphate and copper phosphate) at a certain pH. These complexes cannot be absorbed/taken up by plants [46]. The reduction in plant nutrients such as N, P, and K under the effect of pollution may lead to a reduction in the economic yield and phytomass of almost all vegetable crops [6].

Various abiotic stresses decrease the chlorophyll content in plants [47]. In our study, the reduction of chlorophyll a and b in *P*. *sativum* cultivated in the polluted soils may have been the result of high concentrations of heavy metals, which inhibits chlorophyll synthesis and destroys the chloroplasts [48]. Moreover, *P*. *sativum* was found to accumulate higher concentrations of Cd, Cr, Ni, and Pb. Horler *et al*. [49] studied, in detail, the leaf pigments of *P*. *sativum* plants and showed that chlorophyll a and b decreased under the stress of Cd, Cu, Pb, or Zn. A similar result was obtained by Gubrelay *et al*. [50] in barley.

Heavy metals may inhibit or promote the synthesis of some proteins [51] with a general trend of decline in the overall content. Our study reported that proteins and carbohydrates declined in the tissues of *P*. *sativum* cultivated in polluted soils. This result coincides with the study of Galal [6], who reported a decrease in soluble protein content under heavy metal stress in *Cucurbita pepo*. The reduction in protein content may have been caused by increasing protein degradation as a result of the increased activity of protease enzyme [52], which increases under stress conditions. Gubrelay *et al*. [50] reported a decrease in carbohydrate content in Cd-treated *Oryza sativa* and attributed this to chlorophyll biosynthesis inhibition. Ahmed *et al*. [53] reported that carbohydrates can be inhibited if the Cd concentration is more than 5 mg/kg in *P*. *sativum* tissues. In the present study, the cadmium concentration in the *P*. *sativum* tissues grown in the polluted soils exceeded 100 mg/kg.

The accumulation of metals in vegetables depends on many factors, such as the type of vegetable (leafy, tuber, or fruit), the concentration of metals in the soil, soil organic carbon, and pH. Soil pH plays a significant role in heavy metal investigations, where a low pH enhances the bioavailability of metals [54]. *P*. *sativum* cultivated in polluted soils, with a low pH value, accumulated high concentrations of heavy metals in our study. Nanda and Araham [55] reported that plants accumulating heavy metals exceeding 1000 mg/kg in their tissues were considered to be hyperaccumulators; therefore, *P*. *sativum* is considered a hyperaccumulator for Cd and Fe.

Vegetables absorb metals from contaminated soil and from the particulate matter deposited on their parts from the ambient air [1]. The BF of the studied heavy metals was greater than 1, and this may be a result of the high accumulation power of *P*. *sativum* in relation to these metals [6]. Similar results were reported by Shehata and Galal [9] in Egypt, and by Ali and Al-Qahtani [15] and Eid *et al*. [3] in Saudi Arabia, for some vegetable crops. Chen *et al*. [56] reported that legumes are more likely to accumulate Cr, while leafy vegetables accumulate higher concentrations of Cd and Pb. This is in agreement with our finding that Cd had the highest BF (442.9). In contrast, the TF of the studied heavy metals did not exceed unity in either the nonpolluted or the polluted sites. Therefore, these heavy metals are suitable elements for phytostabilization, which decreases the mobility of metals and their leaching into groundwater; and hence decreases the metal bioavailability and risk of entry into the food chain [9].

Although there are several pathways for human exposure to heavy metals, eating contaminated food is one of the major pathways [57]. The DIM for the assessed heavy metals (except Fe) in the polluted and nonpolluted sites was less than 1 in both children and adults, suggesting that the health risk of single heavy metal exposure through the food chain is generally low [57]. However, Horiguchi *et al*. [58] reported that the amount of ingested of heavy metals is unequal to the absorbed pollutant amount in reality, because the heavy metals ingested may be excreted, with the remainder accumulating in body tissues and affecting human health. According to US-EPA [37], an HRI of >1.0 is considered dangerous for human health. Washington *et al*. [59] showed that human exposure to Cd, Cu, Pb, Zn, and Cr through the food chain is at safe levels because the HRI for heavy metals in vegetables is <1.0. However, the present study indicated that the HRI of Pb, Cd, Fe, and Mn in the polluted sites and Pb in nonpolluted sites was >1.0 by many folds. This will threaten the health of the local population in Egypt, where *P*. *sativum* constitutes a high proportion of the diet. Cadmium is not essential for biological function in humans. The kidney is the main human organ impacted by cadmium exposure in both the general population and in those who are occupationally exposed, while copper is currently categorized by the US-EPA as a Group D carcinogen and can cause the destruction of red blood cells, possibly resulting in anemia [60]. Radwan and Salama [5] reported that Pb, Cd, Cu, and Zn concentrations in fruit and leafy vegetables in the Egyptian markets exceed the permissible limits of heavy metals in the food given by the WHO/FAO [40]. In addition, Hare [61] mentioned that “nonessential” elements such as Cd and Pb, even at low concentrations, are toxic for humans. Therefore, the consumption of *P*. *sativum* poses a high risk to human health, and the cultivation of such an important vegetable in farms exposed to pollution is not desirable.

## Conclusions

According to the Environmental Quality Standards set by the US-EPA, the polluted and nonpolluted soils were in the normal range, and this may be due to the incessant removal of heavy metals by crops cultivated in these sites and the leaching of heavy metals into the soil’s deeper layer. The values of all of the metals (except Cr, Cu, and Ni) were above the tolerable limits recommended by the World Health Organization. The BF of the studied heavy metals was greater than 1, while the TF did not exceed unity in either the nonpolluted or polluted sites. Therefore, these heavy metals are suitable elements for phytostabilization, which decreases the mobility of metals and their leaching into groundwater, and hence decreases the metal bioavailability and its risk of entering into the food chain. The DIM for the assessed heavy metals (except Fe) in the polluted and nonpolluted sites was less than 1 in both children and adults, suggesting that the health risk of single heavy metal exposure through the food chain is generally low. The present study indicated that the HRI of Pb, Cd, Fe, and Mn in the polluted site and Pb in nonpolluted site was >1.0 by many folds. This will threaten the health of the local and global populations, where *P*. *sativum* constitutes a high proportion of the diet worldwide.

## Supporting information

S1 Dataset (XLSX)

## References

[1] Sobukola OP, Dairo OU, Sanni LO, Odunewu AV, Fafiolu BO. Thin layer drying process of some leafy vegetables under open sun. Food Sci Technol Int 2007; 13: 35–40.

[2] Galal TM, Khalafallah AA, Elawa OE, Hassan LM. Human health risks from consuming cabbage (*Brassica oleracea* L. var. *capitata*) grown on wastewater irrigated soil. Int J Phytoremed 2018; 20: 1007–1016. doi: 10.1080/15226514.2018.1452186 30095311

[3] Eid EM, Hussain AA, Taher MA, Galal TM, Shaltout KH, Sewelam N. Sewage sludge application enhances the growth of *Corchorus olitorius* plants and provides a sustainable practice for nutrient recirculation in agricultural soils. J Soil Sci Plant Nutr 2020; 20: 149–159.

[4] Oteef MD, Fawy KF, Abd-Rabboh HS, Idris AM. Levels of zinc, copper, cadmium, and lead in fruits and vegetables grown and consumed in Aseer Region, Saudi Arabia. Environ Monit Assess 2015; 187: 676. doi: 10.1007/s10661-015-4905-8 26446130

[5] Radwan MA, Salama AK. Market basket survey for some heavy metals in Egyptian fruits and vegetables. Food Chem Toxicol 2006; 44: 1273–1278. doi: 10.1016/j.fct.2006.02.004 16600459

[6] Galal TM. Health hazards and heavy metals accumulation by summer squash (*Cucurbita pepo* L.) cultivated in contaminated soils. Environ Monit Assess 2016; 188: 434. doi: 10.1007/s10661-016-5448-3 27344559

[7] Galal TM, Sheded ZA, Hassan LM. Hazards assessment of the intake of trace metals by common mallow (*Malva parviflora* L.) growing in polluted soils. Int J Phytoremed 2019; 21: 1397–1406. 10.1080/15226514.2018.1524842 31648539

[8] Dan A, Oka M, Fujii Y, Soda S, Ishigaki T, Machimura T, Ike M. Removal of heavy metals from synthetic landfill leachate in lab-scale vertical flow constructed wetlands. Sci Tot Environ 2017; 584–585: 742–750. 10.1016/j.scitotenv.2017.01.112 28131455

[9] Shehata HS, Galal TM. Trace metal concentration in planted cucumber (*Cucumis sativus* L.) from contaminated soils and its associated health risks. J Cons Prot Food Safe 2020; 15: 205–217.

[10] Chowdhury AH, Chowdhury T, Rahman A. Heavy metal accumulation in tomato and cabbage grown in some industrially contaminated soils of Bangladesh. J Bangladesh Agri Univ 2019; 17(3):288–294.

[11] Hu S, Liu L, Zuo S, Ali M, Wang Z. Soil salinity control and cauliflower quality promotion by intercropping with five turfgrass species. J Clean Prod 2020; 266:121991.

[12] Hu WY, Zhang YX, Huang B, Teng Y. Soil environmental quality in greenhouse vegetable production systems in eastern China: current status and management strategies. Chemosphere 2017; 170:183–195. doi: 10.1016/j.chemosphere.2016.12.047 27988454

[13] Singh S, Zacharias M, Kalpana S, Mishra S. Heavy metals accumulation and distribution pattern in different vegetables crops. J Environ Chem Ecotoxicol 2012; 24: 170–177.

[14] Bakht J, Khan L, Shafi M. Phytoaccumulation of heavy metals and protein expression by different vegetables collected from various parts of Khyber Pukhtunkhawa Province, Pakistan. Sains Malaysiana 2016; 45: 167–176.

[15] Ali MH, Al-Qahtani KM. Assessment of some heavy metals in vegetables, cereals and fruits in Saudi Arabian markets. Egy J Aquat Res 2012; 38: 31–37.

[16] Eid EM, Alrumman SA, Galal TM, El-Bebany AF. Regression models for monitoring trace metal accumulations by *Faba sativa* Bernh. plants grown in soils amended with different rates of sewage sludge. Sci Rep 2019; 9: 5443. doi: 10.1038/s41598-019-41807-9 30931965 PMC6443791

[17] Dong Q, Fei L, Wang C, Hu S, Wang Z. Cadmium excretion via leaf hydathodes in tall fescue and its phytoremediation potential. Environ Pollut 2019; 252:1406–1411. doi: 10.1016/j.envpol.2019.06.079 31260940

[18] Iglesias-García R, Prats E, Flores F, Amri M, Mikić A, Rubiales D. Assessment of field pea (*Pisum sativum* L.) grain yield, aerial biomass and flowering date stability in Mediterranean environments. Crop Past Sci 2017; 68: 915–923.

[19] Tóth G, Hermannb T, Da Silva MR, Montanarella L. Heavy metals in agricultural soils of the European Union with implications for food safety. Environ Int 2006; 88: 299–309. 10.1016/j.envint.2015.12.017 26851498

[20] Guerra F, Trevizam AR, Muraoka T, Marcante NC, Canniatti-Brazaca SG. Heavy metals in vegetables and potential risk for human health. Sci Agri 2012; 59: 54–60.

[21] Qing X, Yutong Z, Shenggao L. Assessment of heavy metal pollution and human health risk in urban soils of steel industrial city (Anshan), Liaoning, Northeast China. Ecotoxicol Environ Safe 2015; 120: 377–385. 10.1016/j.ecoenv.2015.06.019 26114257

[22] Borah M, Devi A. Effect of heavy metals on *Pisum sativum* Linn. Int J Adv Biol Res 2012; 2: 314–321.

[23] FAOSTAT. Crops. http://www.fao.org/faostat/en/#data/QC (accessed on 20. November 2020).

[24] Elkhatib HA, Gabr SM, El-Keriawy AM. Mathematical aspects of seed production response of pea (*Pisum sativum* L.) to nitrogen and bio-fertilization. J Agri Environ Sci Alex Univ 2007; 6: 218–238.

[25] El-Sherbiny AE, Galal YG, Soliman SM, Dahdouh SM, Ismail MM, Fathy A. Fertilizer nitrogen balance in soil cultivated with pea (*Pisum sativum* L.) under bio and organic fertilization system using 16N stable Isotope. (4th International Conference on Radiation Science and Applications: Taba) 2014.

[26] Allen SE. Chemical analysis of ecological materials. (Blackwell Scientific Publications: London) 1989.

[27] Metzner H, Rauand H, Senger H. Unter suchungen zur synchronisier barteit einzelner pigmentan angel mutanten von chlorella. Planta 1965; 65: 186.

[28] Umbriet WW, Burris RH, Stauffer JF, Cohen PP, Johanse WJ, Lee PG, et al. Monometric technique, a manual description method, applicable to study of desiring metabolism. (Burgess Publishing Company: Minnesota) 1959.

[29] Lowry OH, Rosen BJ, Fan AC, Randel RJ. Protein measurement with Folin phenol reagent. J Biol Chem 1951; 193: 225–275. 14907713

[30] Lu RK. Methods of inorganic pollutants analysis. (Agricultural Science and Technology Press: Beijing) 2000.

[31] Liu WH, Zhao JZ, Ouyang ZY, Soderlund L, Liu GH. Impacts of sewage irrigation on heavy metals distribution and contamination in Beijing, China. Environ Int 2005; 31: 805–812. doi: 10.1016/j.envint.2005.05.042 15979146

[32] SPSS. SPSS base 15.0 users guide. (SPSS Inc.: Chicago) 2006.

[33] Khan S, Farooq R, Shahbaz S, Aziz Khan M, Sadique M. Health risk assessment of heavy metals for population via consumption of vegetables. World Appl Sci J 2009; 6: 1602–1606.

[34] Rattan R, Datta S, Chhonkar P, Suribabu K, Singh A. Long-term impact of irrigation with sewage effluents on heavy metal content in soils, crops and groundwater: a case study. Agri Ecosys Environ 2005; 109: 310–322.

[35] Asgari K, Cornelis WM. Heavy metal accumulation in soils and grains, and health risks associated with the use of treated municipal wastewater in subsurface drip irrigation. Environ Monit Assess 015; 187: 410. doi: 10.1007/s10661-015-4565-8 26050062

[36] Singh S, Nag SK, Kundu SS, Maity SB. Relative intake, eating pattern, nutrient digestibility, nitrogen metabolism, fermentation pattern and growth performance of lambs fed organically and inorganically produced cowpea hay-barley grain diets. Trop Grasslands 2010; 44: 55–61.

[37] US-EPA (United States Environmental Protection Agency). Reference dose (RfD): Description and use in health risk assessments. Background Document 1A, Integrated Risk Information System (IRIS). 2012; (United States Environmental Protection Agency: Washington, DC).

[38] WHO (World Health Organization). Health criteria other supporting information. In Guidelines for drinking water quality. 1996; (World Health Organization: Geneva).

[39] US-EPA (United States Environmental Protection Agency). Reference dose (RfD): Description and use in health risk assessments. Background Document 1A, Integrated Risk Information System (IRIS). 2013; (United States Environmental Protection Agency: Washington, DC).

[40] WHO/FAO (World Health Organization, Food and Agriculture Organization of the United Nations). Guidelines for the safe use of wastewater and food stuff. Wastewater Use in Agriculture. 2013; (World Health Organization: Geneva).

[41] US-EPA (United States Environmental Protection Agency). Risk-based concentration table. 2010; (United State Environmental Protection Agency: Washington, DC).

[42] WHO/FAO (World Health Organization, Food and Agriculture Organization of the United Nations). Evaluation of certain food additives and contaminants. In Sixty-first report of the joint FAO/WHO expert committee on food additives. 2004; (World Health Organization: Geneva).

[43] Chibuike GU, Obora SC. Heavy metal polluted soils: Effect on plants and bioremediation methods. Appl Environ Soil Sci 2014; 752708.

[44] Lionetto MG, Caricato R, Calisi A, Giordano ME, Erroil E, Schettino T. Biomonitoring of water and soil quality: a case study of ecotoxicological methodology application to the assessment of reclaimed agroindustrial wastewaters used for irrigation. Rend Lin 2016; 27: 105–112.

[45] Mansur UD, Garba KA. Effect of some heavy metal pollutants on fertility characteristics of an irrigated savannah Alfisol. Bayero J Pure Appl Sci 2010; 3: 255–259.

[46] Aydinalp C, Marinova S. Distribution and forms of heavy metals in some agricultural soils. Polish J Environ Stud 2003; 12: 629–633.

[47] Kumar V, Awasthi G, Chauhan PK. Cu and Zn tolerance and responses of the biochemical and physiochemical system of wheat. J Stress Physiol Biochem 2012; 8: 203–213.

[48] Zeid IM, Ghazi SM, Nabawy DM. Alleviation of Co and Cr toxic effects on alfalfa. Int J Agron Plant Prod 2013; 4: 984–993.

[49] Horler DN, Barber J, Barringer AR. Effect of heavy metals on the absorbance and reflectance spectra of plants. Int J Rem Sens 1980; 1: 121–136.

[50] Gubrelay U, Agnihotri RK, Singh G, Kaur R, Sharma R. Effect of heavy metal Cd on some physiological and biochemical parameters of Barley (*Hordeum vulgare* L.). Int J Agri crop Sci 2013; 5: 2743–2751.

[51] John R, Ahmad P, Gadgil K, Sharma S. Effect of cadmium and lead on growth, biochemical parameters and uptake in *Lemna polyrrhiza* L. Plant, Soil Environ 2008; 54: 262–270.

[52] Palma JM, Sandalio LM, Javier-Corpas F, Romero-Puertas MC, McCarthy I, del Rio LA. Plant proteases protein degradation and oxidative stress: Role of peroxisomes. Plant Physiol Biochem 2002; 40: 521–530.

[53] Ahmad P, Sharma S, Srivastava PS. Differential physio-biochemical responses of high yielding varieties of Mulberry (*Morusalba*) under alkalinity (Na_2_CO_3_) stress *in vitro*. Physiol Molec Biol Plants 2006; 12: 59–66.

[54] Jhamaria C, Bhatnagar M, Naga JP. Accumulation of heavy metals in soil and vegetables due to wastewater irrigation in a semiarid region of Rajasthan, India. Int J Environ Ecol Family Urban Stud 2015; 5: 1–10.

[55] Nanda S, Abraham A. Remediation of heavy metal contaminated soil. Afr J Biotechnol 2013; 12: 3099–3109.

[56] Chen YW, Shao Y, Ying Y. Health risk assessment of heavy metals in vegetables grown around battery production area. Sci Agri 2014; 71: 126–132.

[57] Zeng X, Wang Z, Wang J, Guo J, Chen X, Zhuangs J. Health risk assessment of heavy metals via dietary intake of wheat grown in Tianjin sewage irrigation area. Ecotoxicol 2015; 24: 2115–2124. doi: 10.1007/s10646-015-1547-0 26433741

[58] Horiguchi H, Oguma E, Sasaki S, Miyamoto K, Ikeda Y, Machida M, et al. Dietary exposure to cadmium at close to the current provisional tolerable weekly intake does not affect renal function among female Japanese farmers. Environ Res 2004; 95: 20–31. doi: 10.1016/S0013-9351(03)00142-7 15068927

[59] Washington DC, Wang Y, Qiao M, Liu Y, Zhu Y. Health risk assessment of heavy metals in soils and vegetables from wastewater irrigated area, Beijing-Tianjin city cluster, China. J Environ Sci 2012; 24: 690–698. 10.1016/s1001-0742(11)60833-4 22894104

[60] Mahurpawar M. Effect of heavy metals on human health. Int J Res Granth 2015; 3: 1–7.

[61] Hare L. Aquatic insects and trace metals: Bioavailability, bioaccumulation and toxicity. Crit Rev Toxicol 1992; 22: 327–369. doi: 10.3109/10408449209146312 1489510

